# Prescribing practices, patterns, and potential harms in patients receiving palliative care: A systematic scoping review

**DOI:** 10.1016/j.rcsop.2021.100050

**Published:** 2021-07-23

**Authors:** Cathal A. Cadogan, Melanie Murphy, Miriam Boland, Kathleen Bennett, Sarah McLean, Carmel Hughes

**Affiliations:** a School of Pharmacy and Pharmaceutical Sciences, Trinity College Dublin, Ireland; b School of Pharmacy and Biomolecular Sciences, Royal College of Surgeons in Ireland, Ireland; c Division of Population Health Sciences, Royal College of Surgeons in Ireland, Ireland; d St Vincent's Private Hospital, Dublin, Ireland; e School of Pharmacy, Queen's University Belfast, United Kingdom

**Keywords:** Palliative care, Prescribing, Scoping review, Observational, Cohort studies

## Abstract

**Background:**

Patients receiving palliative care often have existing comorbidities necessitating the prescribing of multiple medications. To maximize quality of life in this patient cohort, it is important to tailor prescribing of medication for preventing and treating existing illnesses and those for controlling symptoms, such as pain, according to individual specific needs.

**Objective(s):**

To provide an overview of peer-reviewed observational research on prescribing practices, patterns, and potential harms in patients receiving palliative care.

**Methods:**

A systematic scoping review was conducted using four electronic databases (PubMed, EMBASE, CINAHL, Web of Science). Each database was searched from inception to May 2020. Search terms included ‘palliative care,’ ‘end of life,’ and ‘prescribing.’ Eligible studies had to examine prescribing for adults (≥18 years) receiving palliative care in any setting as a study aim or outcome. Studies focusing on single medication types (e.g., opioids), medication classes (e.g., chemotherapy), or clinical indications (e.g., pain) were excluded. The review followed the Preferred Reporting Items for Systematic Reviews and Meta-Analyses (PRISMA) guidelines for scoping reviews, and the findings were described using narrative synthesis.

**Results:**

Following deduplication, 16,565 unique citations were reviewed, and 56 studies met inclusion criteria. The average number of prescribed medications per patient ranged from 3 to 23. Typically, prescribing changes involved decreases in preventative medications and increases in symptom-specific medications closer to the time of death. Twenty-one studies assessed the appropriateness of prescribing using various tools. The prevalence of patients with ≥1 potentially inappropriate prescription ranged from 15 to 92%. Three studies reported on adverse drug events.

**Conclusions:**

This scoping review provides a broad overview of existing research and shows that many patients receiving palliative care receive multiple medications closer to the time of death. Future research should focus in greater detail on prescribing appropriateness using tools specifically developed to guide prescribing in palliative care and the potential for harm.

## 1 Introduction

As a consequence of population aging, there is an ever-increasing demand for palliative care for individuals with limited life expectancy.^1^ Palliative care is defined by the World Health Organization (WHO) as “an approach that improves the quality of life of patients and their families facing the problems associated with life-threatening illness, through the prevention and relief of suffering by means of early identification and impeccable assessment and treatment of pain and other problems, physical, psychosocial and spiritual.”^2^ Historically, palliative care was synonymous with end-of-life care provided through hospices.^3^ Increasingly it is recognized that palliative care is applicable in the early stages of a life-limiting illness, in conjunction with other treatments intended to prolong life, and is not limited to hospice settings.^4^ Therefore, the scope of palliative care encompasses the care provided to individuals from the point of diagnosis of any life-limiting illness through to end of life.^5^ Adequate provision of palliative care is recognized as a major public health issue, and dedicated strategies are required to ensure effective integration of palliative care into healthcare systems.^6^ A key component of any such strategy involves ensuring the availability of necessary medications intended to treat existing conditions and relieve symptoms experienced by patients at the end of life, such as pain, and ensure that they are prescribed appropriately.

Ensuring appropriate prescribing for patients receiving palliative care is a major challenge to improving quality of life and is an under-researched area.^7^, ^8^, ^9^, ^10^ Patients with limited life expectancy often have existing comorbidities necessitating the use of polypharmacy which is commonly defined as the prescribing of five or more medications.^8^^,^^9^ Optimising medication regimens requires clinicians to consider whether each medication is appropriate in relation to patients' context, treatment goals, and life expectancy.^9^^,^^11^^,^^12^ Under these circumstances, the goal of prescribing moves from preventing and treating existing illnesses to controlling symptoms, such as pain, and improving patients' quality of life.

In recent years, discussion regarding opportunities for deprescribing across healthcare settings has been presented, primarily in the context of older adults (≥65 years).^13^ Deprescribing is defined as a systematic process involving identifying and discontinuing medicines in cases where potential or existing harms outweigh benefits.^14^ This process is conducted within the context of the individual patient's care goals, values, preferences, and current level of functioning, and life expectancy.^14^ Previous reviews have examined prescribing for patients with life-limiting illnesses and focused on preventative medications (i.e., chronic medication used to treat or prevent further worsening of a disease state).^10^^,^^15^ This has helped to characterize the commonly prescribed types of preventative medicines, as well as the methods used to identify potentially inappropriate medications (PIMs) and opportunities for deprescribing. However, it remains unclear whether patients were receiving key palliative care medications required for optimal symptom control towards end of life, such as appropriate analgesia.^16^, ^17^, ^18^

This scoping review aimed to provide an overview of observational research on prescribing practices, patterns, and potential harms in patients receiving palliative care. The objectives were:

1. To examine the number and types of medications prescribed (i.e., preventative and symptom-specific medications) for patients receiving palliative care;
2. To investigate the methods used to assess the appropriateness of medication prescribing for patients receiving palliative care;
3. To examine the risk factors/determinants of potentially inappropriate prescribing for patients receiving palliative care;
4. To establish the types of potential harms (i.e., adverse drug events, drug interactions) associated with prescribing for patients receiving palliative care;
5. To examine changes in medication prescribing for patients receiving palliative care over time.

## 2 Material and methods

This scoping review was conducted and reported in accordance with relevant methodological guidance and the Preferred Reporting Items for Systematic Reviews and Meta-Analyses guidelines for scoping reviews (PRISMA-ScR) [Appendix A].^19^^,^^20^ The review protocol is available from the authors on request.

For the purpose of this review, palliative care was defined using the WHO's definition, as outlined in the introduction above.^2^ In order to meet inclusion criteria, studies must have examined medication prescribing for adult patients (≥18 years) receiving palliative care for any life-limiting illness in any setting as a study aim or outcome. This could encompass one or more of the following: (1) assessments of prescribed medications; (2) assessments of the appropriateness of medication prescribing and/or associated risk factors for potential harms; (3) assessments of changes in medication prescribing over time. At a minimum, studies must have provided a summary statistic regarding the number of medications that patients were receiving and information on the types of medication prescribed. Studies that also examined prescribing in patient groups that were not specifically receiving palliative care were eligible for inclusion, provided that data for the palliative care group were reported separately. Eligible study designs consisted of cross-sectional, case-control, and cohort studies. Any assessment of the appropriateness of prescribing was acceptable, including clinicians' professional judgment and validated assessment criteria (e.g., Beers criteria for older adults).^21^ Only full-text manuscripts published in English were eligible for inclusion. Studies were excluded in each of the following instances:

• Case reports and case series studies enrolling ≤10 patients;
• Studies that did not report a summary statistic regarding the number of medications that patients were receiving;
• Studies focusing on single medication types, medication classes, or clinical indications as they did not provide a holistic overview of prescribing practices for sample populations;
• Studies of patients with life-limiting illnesses that were not explicitly receiving palliative care;
• Non-English language publications;
• Published conference abstracts due to a lack of sufficient information.

### 2.1 Search strategy and data extraction

Electronic searches were conducted using PubMed, EMBASE, CINAHL, and Web of Science from the date of inception to May 2020 using established search methods for scoping reviews (Appendix B).^19^ Briefly, preliminary searches of each electronic database were undertaken to identify keywords and index terms for articles relating to the review topic. This informed the development of a comprehensive search strategy developed with the assistance of a research librarian using all identified keywords and index terms for each electronic database. Key search terms included: palliative care, end-of-life care, life-limiting illness, and prescribing. Following completion of the electronic database searches, reference lists of all studies meeting inclusion criteria were screened for additional studies.

All abstracts were screened for inclusion by one author (CC). A 20% sample of abstracts was double screened by a second author (MM). If a study appeared to meet inclusion criteria, full-text articles were retrieved and assessed for inclusion by two authors working independently (CC, MB). Any disagreements were resolved by discussion with other members of the research team.

One author (CC) performed data extraction using a data extraction form that was developed in accordance with relevant methodological guidance.^19^ The data extraction form was piloted on a sample of three included studies and refined accordingly. Data were extracted relating to each of the following key headings:

1. *Study*: Authors, year of publication, country, study design, study setting, study outcomes.
2. *Patients*: Sample size, age, gender, life-limiting illness, other medical conditions.
3. *Prescribing*: Assessment time points, medication burden (number of medicines), preventative medications, medications for symptomatic relief, potentially inappropriate prescriptions and criteria used to assess (if any), medication changes (new and/or discontinued prescriptions).
4. *Potential harms:* Assessment methods and time points, types of ADEs/drug interactions, associated risk factors.

The data extraction process was intended to enable a logical and descriptive summary of the review findings to be presented that aligned with the review objectives.

### 2.2 Quality assessment of included studies

As the aim of a scoping review is to provide a broad overview of the existing literature relating to the research question, formal assessments of the methodological quality of included studies are not routinely undertaken.^19^ However, in summarizing, synthesizing, and interpreting the body of literature identified in this review, critical appraisal was conducted focusing specifically on the generalisability of study findings.

### 2.3 Data analysis and synthesis

Palliative care populations can differ extensively with respect to age, diagnoses, functional status, symptom burden, and survival.^22^ In light of this and observed heterogeneity in previous related reviews,^10^^,^^15^ the findings of this review were described using narrative synthesis, which involved the following key steps.^22^

(1) A preliminary synthesis of the findings of included studies was developed in which study characteristics and findings were tabulated to summarise key information.
(2) Extracted study data were reviewed to explore any relationships in the data.
(3) The review team critically reviewed the findings of the synthesis process in terms of the available evidence and potential limitations of the evidence sources, and any discrepancies and uncertainties identified relating to the review questions.

NVivo QSR 12 was used to manage the extracted data. This involved coding the extracted data under key headings from the data extraction form (outlined above) and performing a content analysis of this data to identify key similarities and differences across included studies.

## 3 Results

### 3.1 Search results

**Fig. 1:**
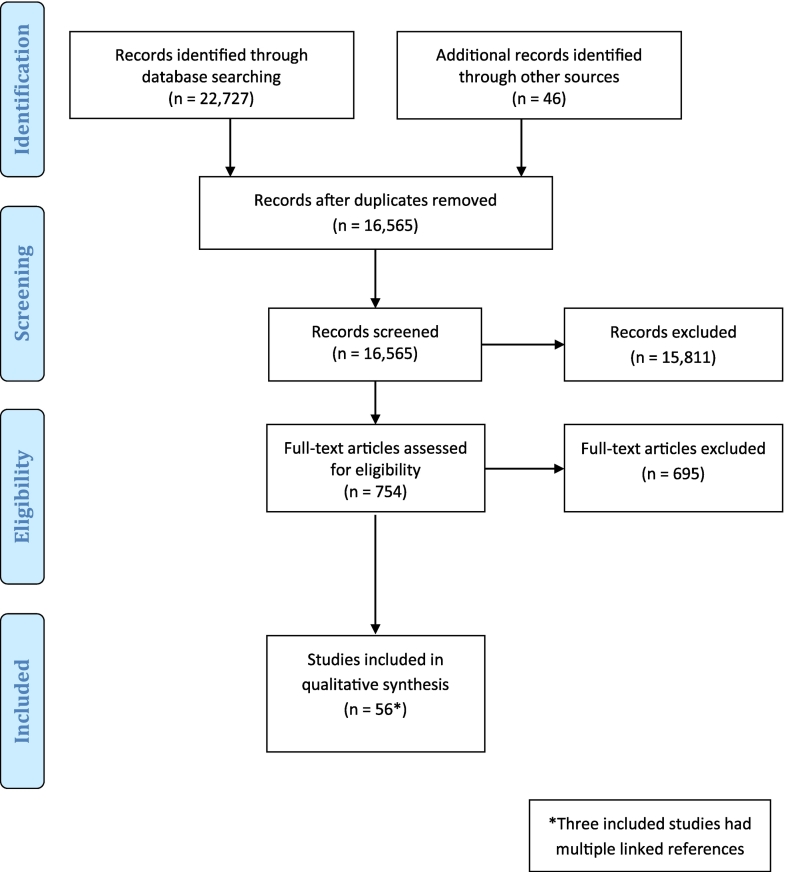
PRISMA flow diagram.

Following deduplication, the electronic searches identified 16,565 unique citations. Following title and abstract screening, 754 full-text articles were reviewed for eligibility. In total, 56 studies met inclusion criteria (Fig. 1).^23^, ^24^, ^25^, ^26^, ^27^, ^28^, ^29^, ^30^, ^31^, ^32^, ^33^, ^34^, ^35^, ^36^, ^37^, ^38^, ^39^, ^40^, ^41^, ^42^, ^43^, ^44^, ^45^, ^46^, ^47^, ^48^, ^49^, ^50^, ^51^, ^52^, ^53^, ^54^, ^55^, ^56^, ^57^, ^58^, ^59^, ^60^, ^61^, ^62^, ^63^, ^64^, ^65^, ^66^, ^67^, ^68^, ^69^, ^70^, ^71^, ^72^, ^73^, ^74^, ^75^, ^76^, ^77^, ^78^, ^79^, ^80^, ^81^ Three studies had more than one reference.^63^^,^^64^^,^^66^^,^^67^^,^^79^^,^^80^ All other articles did not meet the inclusion criteria.

### 3.2 Study design and participants

Table 1 provides an overview of the characteristics of included studies. Study designs consisted primarily of observational cohort studies (52 studies), 14 of which were conducted prospectively. Four studies were based on data collected as part of cross-sectional surveys.^24^^,^^30^^,^^68^^,^^74^ The studies were conducted across 25 countries. Two studies were multinational, involving three and 12 countries, respectively.^56^^,^^58^ Studies were primarily conducted across hospice settings (*n* = 16) and dedicated palliative care centers, units, and/or services (*n* = 22). Other settings included general practice (n = 1), hospitals (*n* = 12), nursing homes (*n* = 3) and long-term care facilities (n = 1). One study was conducted across academic and community-based clinical sites that formed part of a clinical trial led by a palliative care research group. The number of study sites varied (range 1 to 1174), and 27 studies were conducted within a single site.

**Table 1:** Characteristics of included studies.

Study ID	Country	Study design	Setting and number of study sites	Study population	Sample size
Arevalo 2018^23^	Netherlands	Retrospective cohort study	Hospices⁎ 3 sites	54.2% female Mean age (SD): 72.56 (12.57) Most common life-limiting illness: cancer (84.75%)	59
Bercovitz 2008^24^	United States	Cross-sectional survey	Nursing homes 1174 sites	72.6% female Mean age: 80.1 (started palliative care on or prior to admission) or 85.4 (started palliative care after admission) Most common life-limiting illness: heart failure (23.5%)	37,800
Bisht 2008^25^	India	Prospective cohort study	Tertiary hospital Single site	40% female Median age (range): 55 (13–80) Most common life-limiting illness: cancer (100%)	100
Buchanan 2002^26^	United States	Retrospective cohort study	Nursing homes No. of sites not reported	59% female Mean age (SD): 76.4 (13.9) Most common life-limiting illness: cancer (57%)	40,622
Currow 2007^27^	Australia	Prospective cohort study	Regional palliative care program Single program	50% female Mean age (SD): 71 (12) Most common life-limiting illness: cancer (96.5%)	260
Curtis 1993^28^	United States	Retrospective cohort study	Outpatient palliative care service in a tertiary medical center Single site	50.6% female Age not reported Most common life-limiting illness: cancer (100%)	81
Domingues 2015^29^	Portugal	Prospective cohort study	Palliative care unit of a tertiary cancer center Single site	39.4% female Mean age (SD): 68.2 (11.8) Most common life-limiting illness: cancer (100%)	71
Dwyer 2015^30^	United States	Cross-sectional survey	Hospices 1036 sites	54.8% female Age: 65–74 years (19.5%), 75–84 years (36.9%), ≥85 years (43.7%) Most common life-limiting illness: cancer (45.8%)	2623
Foreva 2015^31^	Bulgaria	Prospective cohort study	General practice No. of sites unclear	51.2% female Age: 80% >60 years Most common life-limiting illness: cancer (53.1%)	211
Frechen 2012^32^	Germany	Retrospective cohort study	Hospices 2 sites	54% female Median age (range): 74 (36–99) Most common life-limiting illness: cancer (94%)	364
Garfinkel 2018^33^	Israel	Prospective cohort study	Hospice Single site	49.5% female Mean age (SD): 79.5 (7.9) Most common life-limiting illness: cancer (100%)	202
Grądalski 2019^34^	Poland	Prospective cohort study	Hospice Single site	Gender not reported Mean (SD) age: 74.2 (11.7) Most common life-limiting illness: cancer (95.8%)	337
Hoemme 2019^35^	Switzerland	Retrospective cohort study	Hospital Single site	57.7% female Age: 53.4% ≥65 years Most common life-limiting illness: cancer (100%)	305
Holmes 2008^36^	United States	Prospective cohort study	Long-term care facilities 3 sites	74% female Mean age (range): 83.8 (57–100) Most common life-limiting illness: dementia (100%)	34
Hong 2020^37^	Republic of Korea	Cohort study	Hospital 17 sites	30.9% female Median age (range): 75 (70–93) Most common life-limiting illness: cancer (100%)	301
Hui 2015^38^	United States	Retrospective cohort study	Acute inpatient palliative care unit within a tertiary care cancer center Single site	65% female Mean age (SD): 57.5 (13.2) Most common life-limiting illness: cancer (100%)	100
Jansen 2014^39^	Norway	Retrospective cohort study	Nursing homes 3 sites	59.4% female Median age (range): 86 (19–104) Most common life-limiting illness: dementia (36.8%)	524
Kadoyama 2019^40^	United States	Retrospective cohort study	Tertiary care hospital Single site	46% female Mean age (SD): 65.9 (16.4) Most common life-limiting illness: cancer (49.1%)	348
Khaledi 2019^41^	Iran	Cohort study	Palliative care unit of a hospital Single site	47.8% female Mean age (SD): 55.5 (16.2) Most common life-limiting illness: cancer (100%)	92
Kierner 2016^42^	Austria	Retrospective cohort study	Palliative care ward of a cancer center within a tertiary care university hospital⁎ Single site	62% female Mean age (range): 61.8 (50–71) Most common life-limiting illness: cancer (100%)	50
Kimball 1996^43^	United States	Retrospective cohort study	Not-for-profit home care hospice programmes 3 programmes	2% female Mean age (SD): 39 (8) Most common life-limiting illness: AIDS (100%)	185
Koh 2002^44^	Singapore	Cohort study	3 different palliative care services: 1) Inpatient palliative care consultation service in an acute hospital 2) Inpatient hospice 3) Home care service	48.9% female Age: 59.7% ≥ 65 years Most common life-limiting illness: not reported	345
Kwon 2017^45^	United States	Prospective cohort study	Acute palliative care unit in a tertiary cancer centre Single site	49.8% female Mean age (range): 58 (20–86) Most common life-limiting illness: cancer (100%)	201
Lindsay 2015^46^	Australia	Prospective cohort study	Tertiary hospital Single site	44.3% female Median age (range): 66 (23–93) Most common life-limiting illness: cancer (100%)	61
Lundy 2013^47^	United Kingdom (Northern Ireland)	Retrospective cohort study	Hospices 5 sites	42% female Median age (range): 68 (20–93) Most common life-limiting illness: cancer (91%)	138
Ma 2014^48^	Canada	Retrospective cohort study	Tertiary academic hospitals 2 sites	35.7% female Mean age (SD): 75.9 (12.1) Most common life-limiting illness: cancer (42.9%)	70
Marin 2020^49^	Canada	Retrospective cohort study	University hospital Single site	47% female Age: 82% ≥60 years Most common life-limiting illness: cancer (100%)	266
Masman 2015^50^	Netherlands	Retrospective cohort study	Palliative care centre Single site	50.5% female Median age (IQR): 76 (63–83) Most common life-limiting illness: cancer (88.9%)	208
McLean 2013^51^	Ireland	Retrospective cohort study	Specialist palliative care service comprising an acute hospital and community team Single service	Gender not reported Median age (range): 74.5 (36–91) Most common life-limiting illness: cancer (79%)	52
McNeil 2016^52^	United States	Retrospective cohort study	Academic and community-based clinical sites that formed part of a clinical trial led by a palliative care research group 15 sites	45.1% female Mean age (SD): 74.3 (11.5) Most common life-limiting illness: cancer (51.6%)	244
Mercadente 2001^53^	Italy	Retrospective cohort study	Home palliative care program Single program	44.5% female Mean age (SD): 67.2 (11.7) Most common life-limiting illness: cancer (100%)	128
Molist Brunet 2015^54^	Spain	Prospective cohort study	Acute care unit for older adults within a secondary care hospital Single site	59.9% female Mean age (SD): 86.7 (9.79) Most common life-limiting illness: not reported	87
Molist Brunet 2014^55^	Spain	Cohort study	Acute older adult unit in a secondary care hospital Single site	79.45% female Mean age (SD): 86.1 (5.73) Most common life-limiting illness: dementia (100%)	73
Nauck 2004^56^	Germany, Switzerland, Austria	Retrospective cohort study	Palliative care units 57 sites	52.7% female Mean age (SD): 65.1 (12.8) Most common life-limiting illness: cancer (95.6%)	1304
O'Leary 2018^57^	United States	Retrospective cohort study	Hospital Single site	56.7% female Mean age (SD): 79.1 (± 13.4) Most common life-limiting illness: cancer (36.3%)	430
Paque 2018^58^	Australia, Belgium, Canada, Denmark, Georgia, Germany, Italy, Norway, Portugal, Spain, Switzerland, United Kingdom	Prospective cohort study	Multiple settings that provided palliative care services 24 hospitals, 4 hospices, 1 nursing home, and 1 palliative care home-care service	44% female Mean age (SD): 67.09 (12.51) Most common life-limiting illness: cancer (100%)	720
Pasina 2018^59^	Italy	Retrospective cohort study	Hospice Single site	47.5% female Mean age (SD): 75.3 (12.1) Most common life-limiting illness: cancer (93.9%)	589
Pasina 2020^60^	Italy	Retrospective cohort study	Home palliative care program Single program	49.6% female Median age (IQR): 79.8 (72.5–85.3) Most common life-limiting illness: cancer (91.2%)	1565
Raijmakers 2013^61^	Italy	Retrospective cohort study	Hospice⁎ Single site	38% female Mean age (SD): 72 (14) Most common life-limiting illness: cancer (100%)	60
Riechelmann 2007^62^	Canada	Retrospective cohort study	Ambulatory palliative care service within a hospital Single site	46% female Median age (range): 67 (26–94) Most common life-limiting illness: cancer (100%)	255
Riechelmann 2009^63^^,^^64^	Canada	Retrospective cohort study	Outpatient palliative care clinics within a hospital Single site	49% female Median age (range): 66 (22–94) Most common life-limiting illness: cancer (100%)	372
Roux 2019^65^	France	Retrospective cohort study	University hospital Single site	46.3% female Mean age (SD): 82.1 (8.6) Most common life-limiting illness: cancer (38.3%)	149
Russell 2014^66^^,^^67^	Australia	Prospective cohort study	Two hospice and palliative care services	41.4% female Mean age (SD): 72.9 (12.6) Most common life-limiting illness: cancer (68%)	203
Scholes 1995^68^	United Kingdom (England)	Cross-sectional survey	Home care palliative care services Services provided across three regions	54% female Mean age (range): 67 (28–95) Most common life-limiting illness: cancer (74%)	264
Sera 2014a^69^	United States	Retrospective cohort study	Hospices Single organization across 11 states: number of sites unclear	68.3% female Mean age (SD): 86.4 (10.5) Most common life-limiting illness: failure to thrive or debility (100%)	293
Sera 2014b^70^	United States	Retrospective cohort study	Hospices Single organization across 11 states: number of sites unclear	56.7% female Mean age (SD): 77.5 (14.3) Most common life-limiting illness: cancer (34.6%)	4252
Suhrie 2009^71^	United States	Retrospective cohort study	Palliative care unit for older adults in a medical center Single site	2.2% female Mean age (SD): 79.7 (7.8) Most common life-limiting illness: dementia (39.3%)	89
Tavcar 2014^72^	Slovenia	Retrospective cohort study	Hospital Single site	64% female Mean age (range): 65.6 (43–83) Most common life-limiting illness: cancer (100%)	25
Todd 2014^73^	United Kingdom (England)	Prospective cohort study	Specialist tertiary care palliative care center Single site	48% female Mean age (range): 70 (26–94) Most common life-limiting illness: cancer (82%)	132
Toscani 2009^74^	Italy	Cross-sectional survey	Inpatient palliative care units 53 sites	Gender not reported Mean age (SD): 69 (12) Most common life-limiting illness: cancer (96.8%)	507
Twycross 1994^75^	United Kingdom (England)	Repeated cross-sectional cohort study	Palliative care unit within a hospital Single site	55% female Median age: 70 Most common life-limiting illness: not reported	385 patients over 5 year period (range 58–92 per year)
Van Nordennen 2016^76^	Netherlands	Prospective cohort study	Inpatient palliative care facilities 6 hospices and 1 palliative care unit in a nursing home	43.9% female Mean age (SD): 75 (11.6) Most common life-limiting illness: cancer (81.3%)	155
Wenedy 2019^77^	Singapore	Retrospective cohort study	Hospice Single site	51.1% female Median age (IQR): 73 (62–81) Most common life-limiting illness: cancer (88.8%)	6938
West 2014^78^	Italy	Retrospective cohort study	Hospices⁎ 5 sites	44.9% female Mean age (range): 74 (43–96) Most common life-limiting illness: cancer (100%)	127
Zueger 2018^79^^,^^80^	United States	Retrospective cohort study	Hospices Number of sites not reported	66% female Mean age (SD): 81.3 (8.4) Most common life-limiting illness: cancer (64.3%)	88,957
Zueger 2019^81^	United States	Retrospective cohort study	Hospices Number of sites not reported	67.1% female Mean age (SD): 81.2 (8.4) Most common life-limiting illness: cancer (61.9%)	42,253

⁎ Study also included non-palliative care specific settings.

Sample sizes ranged from 25 to 88,957 patients (Table 1). Four studies involved nationally representative samples of palliative care patients using surveys^24^^,^^30^ or population datasets.^26^^,^^79^ Across included studies, patients' gender profiles varied (25 studies had a majority of female patients, 27 studies had a majority of male patients) with an average age ranging from 39 to 86.7 years. Cancer was the most commonly reported life-limiting illness across studies, with 19 studies focusing specifically on patients with cancer. The time points over which assessments occurred varied and included referral/admission to palliative care and over the last one to two weeks of life (Appendix C). Eleven studies involved cross-sectional assessments of patients receiving palliative care without any clearly identifiable time point.

### 3.3 Prescribing in palliative care

Included studies primarily focused on prescribed medications documented in patients' medical records/charts. Ten studies reported excluding ‘as required’ medication or non-prescription medication (e.g., over-the-counter medications, supplements) from analysis.^27^^,^^37^, ^38^, ^39^^,^^44^^,^^62^^,^^63^^,^^65^^,^^66^^,^^73^ One study specifically focused on off-label medication use.^74^

The average number of medications that patients received at baseline ranged from 3.3–23.3 (Appendix C). Seventeen studies defined the term ‘polypharmacy’ based on either a numerical threshold (twelve studies^29^^,^^33^^,^^34^^,^^37^^,^^41^^,^^42^^,^^47^^,^^52^^,^^54^^,^^55^^,^^76^^,^^77^) or as the prescribing of multiple medications (five studies^25^^,^^32^^,^^44^^,^^51^^,^^58^). Eleven of the 12 studies involving numerical thresholds used a cut-off of five or more medications to define polypharmacy. The remaining study used threshold levels to define the term [‘polypharmacy’ (6–11 drugs), ‘excessive polypharmacy’ (≥12 drugs)].

Thirteen studies categorized medications based on treatment intention (i.e. preventative, symptomatic).^23^^,^^25^^,^^27^^,^^33^^,^^39^^,^^43^^,^^48^^,^^51^^,^^58^^,^^59^^,^^68^^,^^70^^,^^76^ Across these studies, the most commonly reported symptomatic medications were: opioid analgesics, non-opioid analgesics, anxiolytics/hypnotics, anti-emetics, corticosteroids and laxatives. The most commonly reported preventative medications were: antihypertensive agents, anti-thrombotic agents and lipid-modifying agents.

### 3.4 Prescribing appropriateness

Twenty-one studies assessed the appropriateness of prescribing. Summary details of each assessment tool (*n* = 14) are provided in Table 2, which included established tools for assessing appropriate prescribing in the general older population (i.e., Beers criteria, Medication Appropriateness Index), as well as study-specific tools for defined patient populations (e.g., cancer, dementia). The prevalence of patients with ≥1 PIM ranged from 15 to 92% (Table 3). Commonly identified PIMs across studies included lipid-modifying agents, antihypertensives, anti-thrombotic agents, and drugs for peptic ulcer and gastro-oesophageal reflux disease. Four studies examined patient factors associated with PIMs.^34^^,^^37^^,^^46^^,^^80^ One study found that PIMs more commonly occurred in patients who were bed-bound, had the shortest life expectancy, or were discharged from the hospital and admitted to the hospice.^34^ Another study found a significant association between polypharmacy (≥5 medications) and PIM use.^37^ The third study reported various demographics (e.g., increased age, residing in nursing or assisted living facilities) that increased the likelihood of continuing medication with limited benefit after hospice admission.^80^ The remaining study found no patient-specific factors associated with the incidence of PIMs.^42^

**Table 2:** Overview of identified prescribing assessment tools/criteria.

Assessment tool/criteria	Development method	Intended population	Structure	Included studies in which applied
Beers criteria 2003	Delphi method involving 12 experts	Older adults ≥65 years	The criteria are divided across two tables: • Table 1: comprises 48 medications/medication classes to avoid in older adults • Table 2: lists 20 conditions and medications which should be avoided in older adults with these conditions.	Currow 2007^27^
Beers criteria 2012	Delphi method involving 11 experts	Older adults ≥65 years	Consists of 53 medications/medication classes which are divided into three categories: I. Potentially inappropriate medications/medication classes to avoid in older adults II. Potentially inappropriate medications/medication classes to avoid in older adults with certain diseases/syndromes III. Medications to be used with caution in older adults.	Russell 2014^67^
Beers criteria 2015	Delphi method involving 13 experts	Older adults ≥65 years (excluding hospice and palliative care)	Consists of 88 medications/medication classes which are divided into five categories. I. Potentially inappropriate medications/medication classes to avoid in older adults II. Potentially inappropriate medications/medication classes to avoid in older adults with certain diseases/syndromes III. Medications to be used with caution in older adults IV. Potentially clinically important drug-drug interactions to avoid in older adults V. Medications to avoid or the dosage of which should be reduced with varying levels of kidney function in older adults.	Hong 2020^37^
Duplicate prescribing	Not applicable	Patients receiving palliative care (age range not explicitly defined)	Focused on patients receiving ≥2 drugs from any second-level category in the British National Formulary (e.g., duplicate laxatives). The only exception to this was duplicate prescriptions of analgesics, as this was standard practice.	Twycross 1994^75^
Medication Appropriateness Index (MAI) -modified version	Expert panel	Older adults ≥65 years	MAI consists of 10 questions relating to indication, effectiveness, dose, correct direction, practical directions, drug-drug interactions, drug-disease interactions, duplication, duration, and cost. There are three potential response options to each question: (A) appropriate; (B) marginally appropriate; and (C) inappropriate. Each response receives a weighted score. Study-specific modifications to MAI were made. For example, Question 10 (‘Is this drug the least expensive alternative compared to others of equal utility?’) was not included.	Domingues 2015^29^
OncPal deprescribing guideline	Single-phase consensus exercise involving 9 experts	Palliative patients with cancer (age range not explicitly defined)	Consists of eight medication classes (and specific drugs/drug classes within each medication class) which are potentially suitable targets for discontinuation in palliative patients with cancer.	Grądalski 2019^34^; Lindsay 2015^46^; Marin 2020^49^; Wenedy 2019^77^
Palliative Excellence in Alzheimer Care Efforts (PEACE) Programme Criteria	Delphi method involving 12 experts	Patients with advanced dementia for whom palliation of symptoms is the primary therapeutic goal (age range not explicitly defined)	Consists of 69 medications/medication classes divided across four categories: I. Always appropriate II. Sometime appropriate III. Rarely appropriate IV. Never appropriate	Holmes 2008^36^
Study-specific assessment criteria	Details of development not reported (only cites additional literature)	Not explicitly stated	Medications were considered as unnecessary or inappropriate if: (i) time to clinical benefit was longer than remaining survival time; (ii) treatment goals did not align with patients' preferences regarding goals of care, or; (iii) harm posed by treatment outweighed expected benefits.	Grądalski 2019^34^
Study-specific patient-centered prescription assessment model for chronic drug therapy	Not reported	Older adults at end-of-life (age range not explicitly defined)	Multi-level assessment incorporating: I. Patient-centered assessment: to determine patient's global care goal; II. Diagnosis-centered assessment: to classify each drug according to therapeutic purpose (i.e., preventative, symptomatic) and assess alignment with patient's main care goal; III. Medication-centered assessment: to assess high-risk medication; high-risk combinations; poorly tolerated drugs in frail adults; drugs associated with rapid symptomatic decline if stopped; inappropriate doses and therapeutic duplications.	Molist Brunet 2015^54^
Study-specific assessment criteria	Details of development not reported	End-of-life patients receiving hospice care (age range not explicitly defined)	Criteria consisted of three main categories based on a medication's use for symptomatic or preventative effects: I. Potentially avoidable preventative medications: drugs of limited/no value at end-of-life because time to treatment benefit is shorter than remaining life expectancy; II. Medications of uncertain appropriateness: drugs requiring a case-by-case evaluation; III. Potentially appropriate treatments: medications for symptomatic relief.	Pasina 2018^59^; Pasina 2020^60^
Study-specific assessment criteria	International survey involving 20 experts	Patients with cancer during the last three days of life (age range not explicitly defined)	Consists of 12 medication classes classified as potentially inappropriate in patients with cancer during the last three days of life	Raijmakers 2013^61^; West 2014^78^
Study-specific assessment criteria	Details of development not reported	Patients with advanced cancer and solely receiving palliative care (age range not explicitly defined)	Drugs for comorbid illnesses or self-reported symptoms were classified as futile medications if they were considered unnecessary or duplicates. An unnecessary medication was defined as any medication that did not result in significant patient benefit in terms of survival or symptom control; lacked evidence to support its use (e.g., unproven efficacy); or where treatment goals were only expected with long-term chronic use (e.g., statins for hypercholesterolemia).	Riechelmann 2009^63^
Study-specific assessment criteria	List of unnecessary medications identified based on a previous systematic review and list of essential medications identified based on recommendations of three different healthcare organizations. Both lists were reviewed by three clinicians.	Older adults ≥65 years receiving palliative care	List of unnecessary medications comprising 22 drug classes and examples of specific drugs within each class. List of essential medications comprising 20 drug classes and examples of specific drugs within each class.	Roux 2019^65^
Study-specific assessment tool (Unnecessary Drug Use Measure)	Details of development not reported	Palliative care unit for older adults (age range not explicitly defined)	Consists of three items from the Medication Appropriateness Index relating to: I. Lack of indication II. Lack of effectiveness III. Therapeutic duplication	Suhrie 2009^71^
Study-specific assessment tool (adapted from Holmes et al. 2008)	Delphi method involving 10 experts	Day care patients attending a specialist palliative care center (age range not explicitly defined)	Final criteria not reported	Todd 2014^73^
Study-specific assessment criteria	Developed based on published literature	Patients receiving palliative care (age range not explicitly defined)	Lists seven therapeutic drug classes considered to be of limited benefit in patients receiving palliative care and specific drugs/drug classes within each therapeutic drug class, as well as a number of disease-specific exceptions.	Zueger 2018^79^^,^^80^; Zueger 2019^81^

**Table 3:** Assessment of prescribing appropriateness.

Study ID	Assessment tool/criteria	Prevalence of potentially inappropriate prescriptions	Commonly identified potentially inappropriate prescriptions	Changes in potentially inappropriate prescribing over time
Currow 2007^27^	Beers criteria 2003	15% (*n* = 39) of patients with ≥1 potentially inappropriate medications (PIMs) 79% (31/39) of these patients taking high risk PIMs	Not reported	Proportion of high-risk symptom-specific PIMs increased over time (29% to 48%) Proportion of high-risk PIMs for comorbid conditions remained stable (13% to 15%)
Domingues 2015^29^	Medication Appropriateness Index (MAI) -modified version	23% (*n* = 145) of medications did not have a clinical indication in the palliative care setting	Hemostatic agents, lipid-modifying agents, anti-anemic agents, antibiotics (prevalence of individual PIMs not reported)	Not assessed
Grądalski 2019^34^	Combination of OncPal deprescribing guidelines and study-specific assessment criteria	42.1% (*n* = 142) of patients with ≥1 PIM Potential prescribing omissions (PPOs): 31.5% of patients with concomitant drug deficiency (e.g., absence of laxatives in the cases of regular administration of strong opioids) and 2.1% of patients lacking drugs for specific symptoms (i.e., pain, seizures, depression, delirium, thrombosis)	PIMs: Proton pump inhibitors (21%), lipid-lowering agents (9.5%) PPOs: No laxative when opioid administered (24%), no co-analgesics in pain with neuropathic component (11%), no ‘rescue’ drug when regular opioid administered (10.4%)	Not assessed
Holmes 2008^36^	Palliative Excellence in Alzheimer Care Efforts (PEACE) Programme Criteria	29% (*n* = 10) of patients taking a medication considered to be never appropriate 5% of all 221 medications prescribed considered to be never appropriate	Acetylcholinesterase inhibitors, clopidogrel, estrogen, statins (prevalence of individual PIMs not reported)	Not assessed
Hong 2020^37^	Beers criteria 2015	45.5% (*n* = 137) of patients with ≥1 PIM	Megestrol acetate (37.2%), proton pump inhibitors (27.7%), sulfonylurea (25.5%), benzodiazepines (12.4%)	Not assessed
Lindsay 2015^46^	OncPal deprescribing guideline	70% (*n* = 43) of patients with ≥1 PIM 21.4% (*n* = 132) of all medications considered to be PIMs	Antihypertensives (44%), lipid modifying agents (31%), and CAMs (complementary alternative medicines; 31%)	Not assessed
Marin 2020^49^	OncPal deprescribing guideline	82% (*n* = 219) of patients were found to be taking ≥1 PIM prior to palliative care consultation	Vitamins, minerals, and CAM, antihypertensives, gastric protectants (prevalence of individual PIMs at patient-level not reported)	Reduction in the proportion of patients with ≥1 PIM after palliative care consultation (82% to 57%)
Molist Brunet 2015^54^	Study-specific patient-centered prescription assessment model for chronic drug therapy	39.8% (*n* = 123) of patients with ≥1 PIM at baseline	Antithrombotic agents (26.7%), antihypertensives (21.7%), vitamins/mineral supplements (11.7%), lipid modifying agents (10%), anti-diabetic medications (10%)	Not clearly reported: states that during admission, medication regimens were modified in 93.4% of cases with PIMs
Pasina 2018^59^	Study-specific assessment criteria	86.8% (*n* = 511) of patients with ≥1 potentially avoidable preventative medication (PAPM) at hospice admission 53% (*n* = 312) of patients with ≥1 preventative medication of uncertain appropriateness (PMUA) at hospice admission	PAPMs: drugs for peptic ulcer and gastro-oesophageal reflux disease (77.1%), anti-thrombotic agents (32.3%), beta-blockers (18.3%) PMUAs: diuretics (31.2%), antibiotics (13.9%), antifungals (11.7%)	Reduction in proportion of patients with ≥1 PAPM prior to death (86.8% to 48.6%) Reduction in proportion of patients with ≥1 PMUA prior to death (53% to 30.4%)
Pasina 2020^60^	Study-specific assessment criteria	92.1% (*n* = 1441) of patients with ≥1 potentially avoidable preventative medication (PAPM) at baseline 51.3% (*n* = 803) of patients with ≥1 preventative medication of uncertain appropriateness (PMUA) at baseline	PAPMs: drugs for peptic ulcer and gastro-oesophageal reflux disease (77.4%), anti-thrombotic agents (47.5%), beta-blockers (26.9%) PMUAs: diuretics (36.3%), antibiotics (9.3%), anti-asthmatics (6.4%)	Reduction in proportion of patients with ≥1 PAPM prior to death (92.1% to 60.8%) Reduction in proportion of patients with ≥1 PMUA prior to death (51.3% to 38.9%)
Raijmakers 2013^61^	Study-specific assessment criteria	No overall summary statistics regarding the prevalence of PIMs Reports on proportions of patients with particular PIMs over the last three days of life	Corticosteroids (72%), drugs for peptic ulcer and gastro-oesophageal reflux disease (40%), anticoagulants (23%)	Not assessed for hospice population
Riechelmann 2009^63^	Study-specific assessment criteria	22% (*n* = 82) of patients with ≥1 futile medication	Statins (56%), multivitamins (30%)	Reduction in the proportion of patients with ≥1 futile medication (from 22% to 20%) Statins were discontinued in four patients No duplicate medications were discontinued
Roux 2019^65^	Study-specific assessment criteria	91.3% (136) of patients had ≥1 PIM 90 days before death	Anti-thrombotic agents (38.2%) Drugs for acid-related disorders (29.5%)	Reduction in the proportion of patients with ≥1 PIM closer to time to death (91.3% at 90 days before death, 81.2% during the last week of life, and 34.9% on day of death)
Russell 2014^67^	Beers criteria 2012	25.9% (*n* = 157) of PRN prescriptions considered PIMs	Not reported	Not assessed
Suhrie 2009^71^	Study-specific assessment tool (Unnecessary Drug Use Measure)	40.5% (*n* = 36) of patients with a medication that did not have a clinical indication upon admission/transfer to the palliative care unit	Not reported	Reduction in the proportion of patients (40.5% to 20.2%) with a medication that did not have a clinical indication from admission/transfer to palliative care unit to last medication review prior to death
Todd 2014^73^	Study-specific assessment tool (adapted from Holmes et al. 2008)	70% (*n* = 92) of patients with ≥1 PIM 16% (*n* = 238) of all prescribed medications considered to be PIMs	Statins (27%), mineral supplements (24%), aspirin (20.5%), ACE inhibitors (19.6%), beta-blockers (18.9%)	Not assessed
Twycross 1994^75^	Duplicate prescribing	17% (*n* = 66) of patients with duplicate prescriptions over the entire study period and approximately half of these considered acceptable	Examples provided, e.g., diazepam and temazepam (prevalence of individual duplicates not reported)	Longitudinal data presented on prevalence of duplicate prescribing over study years Consistent decreases reported each year (from 21% in 1988 to 12% in 1992)
Wenedy 2019^77^	OncPal deprescribing guideline	23.7% (*n* = 1641) of patients with ≥1 PIM	Senna glycosides (67%), lactulose (59%), omeprazole (52.1%)	Not assessed
West 2014^78^	Assessment criteria previously developed by Raijmakers et al. 2013	84.1% (*n* = 107) of patients with ≥1 PIM	Drugs for peptic ulcer and gastro-oesophageal reflux disease (64.6%), corticosteroids (62.2%), anticoagulants (33.9%)	Reports on proportions of patients with particular PIMs stopped over the last three days of life No overall summary statistics regarding change in prevalence of PIMs
Zueger 2018^79^^,^^80^	Study-specific assessment criteria	78.7% (*n* = 70,035) of patients actively used ≥one limited benefit medication prior to hospice admission	Antihypertensives (50.6%), proton pump inhibitors (31.1%), anti-hyperlipidemics (29.9%)	Reduction in the proportion of patients (78.7% to 23.7%) actively using at least one limited benefit medication prior to hospice admission
Zueger 2019^81^	Study-specific assessment criteria	14.6% (*n* = 6156) of patients receiving ≥one limited benefit medication prior to hospice admission	Antihypertensives (7.4%), proton pump inhibitors (4.5%)	Not assessed

Only one study reported on under-prescribing.^34^ This study reported concomitant drug deficiency (e.g., absence of laxatives in the cases of regular administration of strong opioids) in 31.5% of patients and an absence of drugs for specific symptoms (i.e. pain, seizures, depression, delirium, thrombosis) in 2.1% of patients.

### 3.5 Potential harms

Three studies reported on ADEs (i.e., harms caused by medication use).^37^^,^^54^^,^^57^ One study examined prescribing for end-of-life care patients within an acute care unit for older adults in a secondary care hospital.^54^ On admission, ADEs were identified in 21% of patients. The most commonly identified ADEs were symptomatic hypotension, blood disorders, falls, and hypoglycemia. The study reported a significant positive correlation between the number of prescribed medications and the incidence of ADEs and a significantly higher prevalence of ADEs in patients with inappropriate prescriptions compared to patients with appropriate drug therapy (37.7% vs. 5.35%, *p* < 0.001). However, the severity, causality, and preventability of identified ADEs were not assessed. Another study examined adverse drug reactions (ADRs) in patients receiving palliative care during an inpatient hospital admission over one year.^57^ The study reported that 57.4% of patients experienced at least one ADR. The most commonly affected organ systems were gastrointestinal, neurological, and dermatological. The medications most commonly associated with ADRs were antimicrobials, opioids, and anticoagulants. The remaining study reported on chemotherapy-related toxicity, which was observed in 53.8% of older patients with cancer receiving first-line palliative chemotherapy.^37^ Forty-one percent of patients visited an emergency room or were hospitalized due to chemotherapy-related toxicity. A significant association was identified between polypharmacy (≥5 medications) and hospitalization or emergency room visits in these patients.

Eight studies reported on drug-drug interactions.^32^^,^^34^^,^^35^^,^^37^^,^^59^^,^^60^^,^^63^^,^^73^ Interaction detection relied primarily on different computer software systems which classified drug interactions according to potential severity. In three studies, these software systems were supplemented by reviews and classification by healthcare professionals. The proportion of patients with at least one potential drug interaction ranged from 12% to 64%, with further subcategories according to severity level. Identified risk factors for drug interactions included advanced age, presence of comorbid illness, and an increasing number of medications. One study examined the prognostic impact of potential drug interaction on overall survival in patients with advanced cancer receiving palliative care.^35^ This study found that major-risk drug interactions were not significantly associated with overall survival in the study population. Another study involving older patients with cancer receiving first-line palliative chemotherapy reported no significant association between drug interactions and chemotherapy-related toxicity.^37^

### 3.6 Prescribing changes over time

Thirty-two studies reported on changes in prescribing over time (Appendix C). Reported details of the prescribing changes varied, with some studies reporting on specific types of medications and others focusing more broadly on changes in the numbers of medications prescribed. The time points over which changes were assessed and reported also varied, which precluded a detailed synthesis. Commonly reported assessment time points included: during transition to palliative care, from admission/referral to palliative care to death, and over the last one to two weeks of life. Typically, prescribing changes involved decreases in preventative medications and increases in medications for symptom control as the time of death approached.

Two studies examined prescribing trends longitudinally using repeated cross-sectional analyses.^75^^,^^79^ Twycross et al. reported changes in the most commonly prescribed medications within a single palliative care unit between 1988 and 1992.^75^ Morphine and co-danthrusate were consistently identified as the most commonly prescribed medications across all study years. Zueger et al. used a nationally representative population database to examine the most commonly dispensed medications to patients as part of a health insurance program (Medicare Part D) after hospice admission between 2008 and 2013.^79^ The study reported little observed variation in the prevalence of the preventative drug classes (e.g., lipid-modifying agents, antihypertensive agents) examined.

Across the eleven studies that examined changes in the prevalence of PIMs over time, decreases in the prevalence of PIMs were typically reported as death approached (Table 3). One notable exception to this was the study by Currow et al.,^27^ which used Beers criteria to assess both symptom-specific medications and medications for comorbid conditions. The study found that over the assessment period (from patient referral to the palliative care service until death) the proportion of high-risk, symptom-specific PIMs increased (29% to 48%) whereas the proportion of high-risk PIMs for comorbid conditions remained stable (13% to 15%).

## 4 Discussion

This review provides an overview of existing observational research on prescribing practices, patterns, and potential harms in patients receiving palliative care. The broad, scoping nature of the review was intended to overcome limitations of previous reviews, which focused solely on preventative medications among patients with any life-limiting illness irrespective of the type of care received.^10^^,^^15^ Despite the inclusion of 56 studies, the review highlights a limited assessment of prescribing appropriateness, potential harms, and prescribing trends across included studies.

### 4.1 Prescribing in palliative care

The review shows that many patients with palliative care needs received a considerable number of medications at various time points towards the end of life. A number of studies referred to the term ‘polypharmacy,’ which has been widely discussed in the context of prescribing for the general older population.^82^ The studies mirrored previously used definitions for the older population in terms of the numerical thresholds and overall variation.^83^ However, a key challenge in critically reviewing the numbers of medications prescribed was that studies often did not clearly differentiate according to treatment intention (i.e., preventative versus symptomatic relief) or examine prescribing changes over time. One study did find that the total number of medications increased closer to death due to the continuation of medications for comorbid conditions and the addition of symptom-specific medications.^51^ This highlights the importance of classifying medications according to treatment intention in order to review the medications prescribed critically. It is also important to recognize that the number of medications is only one factor contributing to overall treatment burden (i.e., the work that patients must do to take care of their health).^84^ Other medication-related factors that may exacerbate treatment burden include challenges with taking the medication due to the complexity of treatment regimens and any medication-related side effects.

### 4.2 Prescribing appropriateness

The importance of ensuring appropriate prescribing in patients with limited life expectancy is increasingly recognized.^85^^,^^86^ Various frameworks, tools, and classification systems have been developed to assist in identifying potentially unnecessary or futile medications towards end of life.^87^, ^88^, ^89^ For example, Morin et al. have developed a classification system to assess whether drugs are adequate, questionable, or inadequate for older adults at the end of life.^89^ Of the included studies that examined the appropriateness of prescribing, several studies used criteria that were not specifically developed or validated with a palliative care population in mind. For example, three studies used previous versions of Beers criteria.^27^^,^^37^^,^^67^ However, the most recent versions of the criteria state that they are not intended for patients in hospice and palliative care settings.^21^^,^^90^ The challenge with using such tools in palliative care is that they may misclassify medications as PIMs where the medication may have an important role in controlling symptoms for patients with limited life expectancy. For example, Beers criteria recommend that non-steroidal anti-inflammatory drugs should be avoided in older adults due to the risk of gastrointestinal bleeding. However, these drugs can be of particular benefit in treating various forms of cancer pain (e.g., metastatic bone pain).^91^

Progress has been made in developing tools that focus on prescribing in palliative care. For example, the OncPal deprescribing guideline^46^ and PEACE Programme criteria^36^ have been developed to guide prescribing in palliative care for patients with cancer and dementia, respectively. However, existing tools are primarily focused on the deprescribing of unnecessary medications. This is evidenced by the review findings whereby the reported cases of potentially inappropriate prescribing primarily involved medications that were deemed inappropriate or futile. There is growing evidence to support the discontinuation of preventative medications, such as statins, towards end of life.^92^ However, it is important to recognize that the concept of potentially inappropriate prescribing is broader than overprescribing (i.e., prescribing where no clinical indication exists) and misprescribing (i.e., prescribing incorrect doses, frequencies, or durations of treatment that significantly increase the risk of adverse events).^93^ It also includes underprescribing (i.e., the omission of medications for specific clinical indications aimed at prevention or treatment). This is an important issue as patients with palliative care needs experience variable levels of symptoms, and underprescribing of analgesics and other symptom-specific medications has been documented in palliative care populations.^94^, ^95^, ^96^, ^97^, ^98^, ^99^ This was evident in the only included study that reported on underprescribing which encompassed concomitant drug deficiency (e.g. absence of laxatives in the cases of regular administration of strong opioids) and an absence of drugs for specific symptoms (e.g. pain).^34^ However, exact details of how underprescribing was assessed were not reported and no formal assessment tool was cited. The International Association of Hospice and Palliative Care previously developed a list of essential medicines for treating commonly encountered symptoms in palliative care.^17^ However, this list is intended to guide decisions regarding medication availability for palliative care within healthcare systems in satisfying the healthcare needs of the population as opposed to the appropriateness of the individual medications for use in specific populations (e.g. older patients with advanced cancer).

### 4.3 Potential harms

In addition to the limited number of assessments of prescribing appropriateness, only three studies examined ADEs, all of which focused on older adults receiving palliative care in inpatient settings.^37^^,^^54^^,^^57^ The findings were consistent with research into medication-related harms in the general older population, whereby a higher risk was associated with an increasing number of medications.^100^ It remains unclear how changes in patients' medication profile towards the end of life impact the potential for harm, particularly in terms of the addition of medications for symptomatic relief. The included studies that examined drug interactions highlighted considerable risks of harm.^32^^,^^34^^,^^35^^,^^37^^,^^59^^,^^60^^,^^63^^,^^73^ However, further research is required to determine the extent to which these risks translate into actual harm as the only two studies that examined the impact of drug interactions on clinical outcomes found that they were not associated with chemotherapy-related toxicity^37^ or overall survival in patients with cancer receiving palliative care.^35^

### 4.4 Going forward

In advancing research into prescribing in palliative care, it would be important to consider how the synthesis and generalisability of study findings could be enhanced. There were considerable challenges in providing a meaningful synthesis of included studies due to observed heterogeneity. For example, there was variation in terms of the study populations, settings, assessment time points, and information reported for key outcomes of interest to the review, which impacts the applicability and generalisability of the review findings. This is a recognized issue in palliative care research.^22^^,^^101^, ^102^, ^103^ For example, an international multicentre study of palliative care centers across Europe identified wide variation in terms of both the services provided and patients receiving care.^103^ A basic dataset of patient characteristics and medical variables to describe a palliative care cancer population has been developed to standardize reporting.^22^ This tool has undergone pilot testing, and there is scope for adapting it to include details of other medical conditions.^104^ Many of the studies were also limited by their single-site design. Population datasets or clinical registries may help to provide more generalizable findings with the potential to examine longitudinal trends over time.^105^

It is perhaps unsurprising that cancer was the most common diagnosis across included studies, given the historical focus on cancer in palliative care. However, it is important to look at palliative care requirements in other patient populations to avoid the potential for care inequalities previously reported.^106^^,^^107^ Further work is needed to examine the appropriateness of prescribing in palliative care. Additional tools may need to be developed or adapted for other populations with life-limiting conditions (e.g., organ failure). Any developed tools should also include assessments of the appropriateness of medications for symptomatic relief and provide a method for systematically and reliably assessing potential under-prescribing/omissions of any such medications.

### 4.5 Strengths and limitations

This is the first known scoping review of observational research examining prescribing in palliative care. It provides a broad overview of existing published literature and followed rigorous methods. It identified a sizeable number of studies conducted across 25 countries. However, it must be acknowledged that other studies closely related to the review topic may have been excluded because of the review's exclusion criteria (e.g., population datasets that examined prescribing in the last year of life irrespective of the care received).^108^ The inclusion/exclusion criteria were developed to answer the review questions and identify a body of literature that would enable a meaningful synthesis to be provided. The review focused on prescribing of medications for palliative care patients. However, it must be noted that there are other aspects of care towards end of life that may be inappropriate (e.g., diagnostic testing).^109^ Additional limitations were that the review only looked at studies published in the English language, and no grey literature searches were conducted, which may have introduced the potential for publication bias.

## 5 Conclusions

This scoping review provides a broad overview of observational studies examining prescribing in palliative care. The review shows that many patients with palliative care needs receive considerable numbers of medications, including preventative medications that may provide limited or no therapeutic benefit closer to death. A limited number of studies examined the appropriateness of prescribing or the potential for harm. Future research should look to include assessments of prescribing appropriateness using tools that have been developed specifically to guide prescribing in palliative care. This should also include assessments of the appropriateness of medications to relieve common symptoms experienced by palliative care populations towards the end of life.

## Author contributions

CC led on the design of the review protocol and the conduct and writing of the review. MM and MB contributed to study identification. CH, KB, and SMcL each contributed to the development of the review protocol and provided relevant clinical and/or methodological expertise. All authors reviewed drafts of the review and approved the final submission.

## Funding

Cathal Cadogan was jointly supported by the 10.13039/501100001593Irish Cancer Society and the All Ireland Institute of Hospice and Palliative Care [grant number: PAL17CAD].

Melanie Murphy was supported by a Clement Archer Scholarship from the School of Pharmacy and Biomolecular Sciences, 10.13039/100012921Royal College of Surgeons in Ireland.

Miriam Boland was supported by Research Summer School funding from the 10.13039/100012921Royal College of Surgeons in Ireland.

Kathleen Bennett was supported by a 10.13039/100010414Health Research Board award [grant number: RL-15-1579].

The above funders had no involvement in this review.

## Declaration of Competing Interest

None.

## Appendix A Supplementary data

Supplementary material. Image 1
