# Supplementary material for: Prescribing practices, patterns, and potential harms in patients receiving palliative care: A systematic scoping review
Source: Explor Res Clin Soc Pharm. 2021 Jul 23;3:100050. doi: 10.1016/j.rcsop.2021.100050 (PMC9031741; doi:10.1016/j.rcsop.2021.100050)
Supplement: Supplementary file 1 — Supplementary material. [file mmc1.docx]

## Appendices

### Appendix A: Preferred Reporting Items for Systematic reviews and Meta-Analyses extension for Scoping Reviews (PRISMA-ScR) Checklist

| **SECTION** | **ITEM** | **PRISMA-ScR CHECKLIST ITEM** | **REPORTED ON PAGE #** |
| --- | --- | --- | --- |
| **TITLE** | | | |
| Title | 1 | Identify the report as a scoping review. | 1 |
| **ABSTRACT** | | | |
| Structured summary | 2 | Provide a structured summary that includes (as applicable): background, objectives, eligibility criteria, sources of evidence, charting methods, results, and conclusions that relate to the review questions and objectives. | 2 |
| **INTRODUCTION** | | | |
| Rationale | 3 | Describe the rationale for the review in the context of what is already known. Explain why the review questions/objectives lend themselves to a scoping review approach. | 3-4 |
| Objectives | 4 | Provide an explicit statement of the questions and objectives being addressed with reference to their key elements (e.g., population or participants, concepts, and context) or other relevant key elements used to conceptualize the review questions and/or objectives. | 4 |
| **METHODS** | | | |
| Protocol and registration | 5 | Indicate whether a review protocol exists; state if and where it can be accessed (e.g., a Web address); and if available, provide registration information, including the registration number. | 4 |
| Eligibility criteria | 6 | Specify characteristics of the sources of evidence used as eligibility criteria (e.g., years considered, language, and publication status), and provide a rationale. | 4-5 |
| Information sources* | 7 | Describe all information sources in the search (e.g., databases with dates of coverage and contact with authors to identify additional sources), as well as the date the most recent search was executed. | 5 |
| Search | 8 | Present the full electronic search strategy for at least 1 database, including any limits used, such that it could be repeated. | Appendix B |
| Selection of sources of evidence† | 9 | State the process for selecting sources of evidence (i.e., screening and eligibility) included in the scoping review. | 5 |
| Data charting process‡ | 10 | Describe the methods of charting data from the included sources of evidence (e.g., calibrated forms or forms that have been tested by the team before their use, and whether data charting was done independently or in duplicate) and any processes for obtaining and confirming data from investigators. | 6-7 |
| Data items | 11 | List and define all variables for which data were sought and any assumptions and simplifications made. | 6 |
| Critical appraisal of individual sources of evidence§ | 12 | If done, provide a rationale for conducting a critical appraisal of included sources of evidence; describe the methods used and how this information was used in any data synthesis (if appropriate). | N/A |
| Synthesis of results | 13 | Describe the methods of handling and summarizing the data that were charted. | 6-7 |
| **RESULTS** | | | |
| Selection of sources of evidence | 14 | Give numbers of sources of evidence screened, assessed for eligibility, and included in the review, with reasons for exclusions at each stage, ideally using a flow diagram. | 7, Figure 1 |
| Characteristics of sources of evidence | 15 | For each source of evidence, present characteristics for which data were charted and provide the citations. | 7-8, Table 1 |
| Critical appraisal within sources of evidence | 16 | If done, present data on critical appraisal of included sources of evidence (see item 12). | N/A |
| Results of individual sources of evidence | 17 | For each included source of evidence, present the relevant data that were charted that relate to the review questions and objectives. | 7-10, Table 1-2, Appendix C |
| Synthesis of results | 18 | Summarize and/or present the charting results as they relate to the review questions and objectives. | 7-10, Table 1-2, Appendix C |
| **DISCUSSION** | | | |
| Summary of evidence | 19 | Summarize the main results (including an overview of concepts, themes, and types of evidence available), link to the review questions and objectives, and consider the relevance to key groups. | 31 |
| Limitations | 20 | Discuss the limitations of the scoping review process. | 34 |
| Conclusions | 21 | Provide a general interpretation of the results with respect to the review questions and objectives, as well as potential implications and/or next steps. | 34-35 |
| **FUNDING** | | | |
| Funding | 22 | Describe sources of funding for the included sources of evidence, as well as sources of funding for the scoping review. Describe the role of the funders of the scoping review. | 36 |

### Appendix B: PubMed search strategy

| **Line number** | **Search term** |
| --- | --- |
|  | "Palliative Medicine"[Mesh] |
|  | "Palliative Care"[Mesh] |
|  | "Hospice Care"[Mesh] |
|  | "Terminal Care"[Mesh] |
|  | "Hospices"[Mesh] |
|  | Palliative[title/abstract] |
|  | “end of life“[title/abstract] |
|  | “advanced illness”[title/abstract] |
|  | “advanced disease”[title/abstract] |
|  | “life expectancy”[title/abstract] |
|  | “life limiting”[title/abstract] |
|  | “end stage”[title/abstract] |
|  | hospice[title/abstract] |
|  | 1 OR 2 OR 3 OR 4 OR 5 OR 6 OR 7 OR 8 OR 9 OR 10 OR 11 OR 12 OR 13 |
|  | "Inappropriate Prescribing"[Mesh] |
|  | "Drug Prescriptions"[Mesh] |
|  | "Potentially Inappropriate Medication List"[Mesh] |
|  | "Deprescriptions"[Mesh] |
|  | Prescribing[title/abstract] |
|  | Prescript*[title/abstract] |
|  | polypharmacy[title/abstract] |
|  | Medication*[title/abstract] |
|  | Medicine*[title/abstract] |
|  | “potentially inappropriate”[title/abstract] |
|  | "medication appropriateness”[title/abstract] |
|  | Deprescri*[title/abstract] |
|  | Underprescri*[title/abstract] |
|  | Overprescri*[title/abstract] |
|  | 15 OR 16 OR 17 OR 18 OR 19 OR 20 OR 21 OR 22 OR 23 OR 24 OR 25 OR 26 OR 27 OR 28 |
|  | "epidemiologic studies"[MeSH Terms] |
|  | "case-control studies"[MeSH Terms] |
|  | "case-control"[All Fields] |
|  | "cohort studies"[MeSH Terms] |
|  | "cohort"[All Fields] |
|  | "Follow up"[All Fields] |
|  | "observational study"[Publication Type] |
|  | "observational studies as topic"[MeSH Terms] |
|  | "observational study"[All Fields] |
|  | Longitudinal[All Fields] |
|  | "retrospective studies"[MeSH Terms] |
|  | "retrospective"[All Fields] |
|  | "cross-sectional studies"[MeSH Terms] |
|  | "cross-sectional"[All Fields] |
|  | 30 OR 31 OR 32 OR 33 OR 34 OR 35 OR 36 OR 37 OR 38 OR 39 OR 40 OR 41 OR 42 OR 43 |
|  | 14 AND 29 AND 44 |

### Appendix C: Additional characteristics of included studies

| **Study ID** | **Setting** | **Assessment time points** | **Baseline number of medications** | **Most commonly prescribed medications/medication classes** | **Prescribing appropriateness** | **Prescribing changes** |
| --- | --- | --- | --- | --- | --- | --- |
| Arevalo 2018 ^[^[^23^](#_ENREF_23)^]^ | Hospices* | Last week of life | Median: 9 | Opioids (98.3%)  Hypnotics/sedatives or anxiolytics (93.2%)  Drugs for constipation (78.9%)  Non-opioid analgesics and antipyretics (71.1%)  Drugs for peptic ulcer and GORD (64.4%) | Not assessed | Assessed |
| Bercovitz 2008 ^[^[^24^](#_ENREF_24)^]^ | Nursing homes | No clearly identifiable time point (cross-sectional assessment) | Mean: 10 | Pain relief (74.1%)  GI drugs (72.4%)  CNS drugs (68.2%)  Cardiovascular/renal drugs (59.7%)  Metabolic/nutrients (57%) | Not assessed | Not assessed |
| Bisht 2008 ^[^[^25^](#_ENREF_25)^]^ | Tertiary hospital | No clearly identifiable time point (cross-sectional assessment) | Mean (SD): 8.7 (0.38) | Gastrointestinal drugs (99%)  Analgesics (92%)  Antineoplastic/immunomodulating agents (57%)  Systemic antibiotics (41%)  Drugs for the respiratory system (20%) | Not assessed | Not assessed |
| Buchanan 2002 ^[^[^26^](#_ENREF_26)^]^ | Nursing homes | Admission | Mean (SD): 8.1 (4.4) | Diuretic medications (24.9%)  Antidepressants (20.1%)  Anti-anxiety medications (17.4%)  Antipsychotics (8.8%)  Hypnotics (6.1%) | Not assessed | Not assessed |
| Currow 2007 ^[^[^27^](#_ENREF_27)^]^ | Regional palliative care program | Admission/referral to death | Mean (SD): 4.9 (2.9) | Morphine  Paracetamol  Dexamethasone  Fentanyl  Metoclopramide  [exact prevalence not reported] | Assessed (Beers criteria 2003) | Assessed |
| Curtis 1993 ^[^[^28^](#_ENREF_28)^]^ | Outpatient palliative care service in a tertiary medical centre | No clearly identifiable time point (cross-sectional assessment) | Median (range): 5 (0-13) | Analgesics (88%)  Laxatives (65%)  Anti-emetics (51%)  OTC antacids (51%)  H_2_ antagonists (40%)  Corticosteroids (28%) | Not assessed | Not assessed |
| Domingues 2015 ^[^[^29^](#_ENREF_29)^]^ | Palliative care unit of a tertiary cancer centre | Transition to palliative care | Mean: 8.57 | Analgesics (19.2%)  Psychoactive drugs (14.1%)  Antihypertensive agents (8.1%)  Antacids (7.8%)  Hormonal drugs (6.8%) | Assessed (Medication Appropriateness Index (MAI) -modified version) | Assessed |
| Dwyer 2015 ^[^[^30^](#_ENREF_30)^]^ | Hospices | Last week of life | Mean: 10.2 | Analgesics (98%)  Antiemetic and anti-vertigo medications (78%)  Anxiolytics, sedatives, and hypnotics (76%)  Anticonvulsants (71%) | Not assessed | Not assessed |
| Foreva 2015 ^[^[^31^](#_ENREF_31)^]^ | General practice | No clearly identifiable time point (cross-sectional assessment) | Mean (SD): 3.56 (0.06) | Symptomatics (not defined)  ACE inhibitors  Non-opioid analgesics  Beta-blockers  Nitrates  [exact prevalence not reported] | Not assessed | Not assessed |
| Frechen 2012 ^[^[^32^](#_ENREF_32)^]^ | Hospices | Last two weeks of life | Median (range): 10 (1-24) | Morphine (86%)  Lorazepam (78%)  Dipyrone (67%)  Fentanyl (46%)  Metoclopramide (46%)  Macrogol (44%) | Not assessed | Not assessed |
| Garfinkel 2018 ^[^[^33^](#_ENREF_33)^]^ | Hospice | Admission | Mean (SD): 9.2 (3.7) | Opioids (78.7%)  Benzodiazepines (74.3%)  Antihypertensives (60.4%)  Proton pump inhibitors (43.6%)  Metoclopramide (33.7%) | Not assessed | Not assessed |
| Grądalski 2019 ^[^[^34^](#_ENREF_34)^]^ | Hospice | On admission to palliative care and during first palliative care consultation | Median: 7 | Not specifically reported | Assessed (OncPal deprescribing guideline and study-specific implicit criteria) | Assessed |
| Hoemme 2019 ^[^[^35^](#_ENREF_35)^]^ | Hospital | At initiation of systemic first-line palliative treatment or referral to palliative care unit | Median (range): 5 (0–15) | NSAIDs (44.3%)  PPIs (43.3%)  Opiates (28.5%) | Not assessed | Not assessed |
| Holmes 2008 ^[^[^36^](#_ENREF_36)^]^ | Long-term care facilities | No clearly identifiable time point (cross-sectional assessment) | Mean (SD): 6.5 (2.7) | Cardiovascular drugs  Non-narcotic analgesics  Laxatives  Vitamins  Antipsychotic agents  [exact prevalence not reported] | Assessed (Palliative Excellence in Alzheimer Care Efforts (PEACE) Programme Criteria) | Not assessed |
| Hong 2020 ^[^[^37^](#_ENREF_37)^]^ | Hospital | Before beginning first-line palliative chemotherapy | Mean (SD): 4.71 (3.1) | Gastrointestinal medication (53.5%)  Cardiovascular medication (52.8%)  Endocrine medication (42.5%)  Analgesic medication (32.9%). | Assessed (Beers criteria 2015) | Not assessed |
| Hui 2015 ^[^[^38^](#_ENREF_38)^]^ | Acute inpatient palliative care unit within a tertiary care cancer centre | Multiple (immediately before and after palliative care inpatient consultation team; admission to acute palliative care unit; discharge from acute palliative care unit or death) | Mean (SD): 9.2 (4.5) | Antiulcer agents (80%)  Anti-infective agents (71%)  Analgesics (70%)  Laxatives (53%) | Not assessed | Assessed |
| Jansen 2014 ^[^[^39^](#_ENREF_39)^]^ | Nursing homes | Last two weeks of life | Palliative medications   - Median (range): 3 (0-8)   Curative/preventative medications   - Median (range): 3 (0-4) | Opioids (82.6%)  Hypnotics (70.4%)  Antipsychotics (51.1%)  Laxatives (32.6%)  Anxiolytics (30.9%) | Not assessed | Assessed |
| Kadoyama 2019 ^[^[^40^](#_ENREF_40)^]^ | Tertiary care hospital | On discharge from hospital to hospice | Mean (SD): 7.1 (4.8) | Opioids (82.5%)  Anxiolytics/sedatives (62.9%)  Laxatives (57.5%)  Anti-emetics (54.3%)  Non-opioid analgesics (45.4%) | Not assessed | Assessed |
| Khaledi 2019 ^[^[^41^](#_ENREF_41)^]^ | Palliative care unit of a hospital | On referral to palliative care unit | Mean (SD): 7.7 (3.2) | Antacids (75%)  Opioids (75%)  Antibiotics/Antifungals (56.5%)  Laxatives (47.8%)  Anticoagulants (44.6%) | Not assessed | Not assessed |
| Kierner 2016 ^[^[^42^](#_ENREF_42)^]^ | Palliative care ward of a cancer centre within a tertiary care university hospital* | Last nine days of life | Median (IQR): 11 (9-13) | Opioids (94%)  Psychoactive drugs (88%)  Anti-thrombotic agents (88%)  Non-opioid analgesics (70%) | Not assessed | Assessed |
| Kimball 1996 ^[^[^43^](#_ENREF_43)^]^ | Not-for-profit home care hospice programmes | Last two weeks of life | Mean (SD): 12.2 (5) | Antifungals (77%)  Benzodiazepines (75%)  Intravenous opioids (74%)  Paracetamol (66%)  Antibacterials (53%) | Not assessed | Not assessed |
| Koh 2002 ^[^[^44^](#_ENREF_44)^]^ | 3 different palliative care services: (1) Inpatient palliative care consultation service in an acute hospital; (2) In-patient hospice; (3) Home care service | Transition to palliative care | Median (range): 5 (0-11) | Analgesics (55.7%)  Laxatives (50.4%)  Anti-ulcer therapy (42.6%)  Health supplements/vitamins (27%)  Haematinics (21.7%) | Not assessed | Assessed |
| Kwon 2017 ^[^[^45^](#_ENREF_45)^]^ | Acute palliative care unit in a tertiary cancer centre | Admission/transfer to palliative care unit | Off-label prescribing events   - Mean: 11 | Haloperidol (12%)  Hydromorphone (11%)  Morphine (8%)  Lorazepam (6%)  Chlorpromazine (5%)  Dexamethasone (5%) | Not assessed | Not assessed |
| Lindsay 2015 ^[^[^46^](#_ENREF_46)^]^ | Tertiary hospital | No clearly identifiable time point (cross-sectional assessment) | Median (range): 10 (4-21) | Not specifically reported | Assessed (OncPal deprescribing guideline) | Not assessed |
| Lundy 2013 ^[^[^47^](#_ENREF_47)^]^ | Hospices | Multiple time points (admission, discharge, death) | Mean (range): 8 (0-17) | Not specifically reported | Not assessed | Assessed |
| Ma 2014 ^[^[^48^](#_ENREF_48)^]^ | Tertiary academic hospitals | Multiple time points (admission, last week of life or prior to transfer to palliative care unit) | Comfort medications   - Mean: 6   Non-comfort medications   - Mean: 7 | Antibiotics  Anticoagulants  Antacids  Analgesics  Laxatives  [exact prevalence not reported] | Not assessed | Assessed |
| Marin 2020 ^[^[^49^](#_ENREF_49)^]^ | University hospital | Prior to and after palliative care consultation | Mean: 12.3 | Not specifically reported | Assessed (OncPal deprescribing guideline) | Assessed |
| Masman 2015 ^[^[^50^](#_ENREF_50)^]^ | Palliative care centre | Admission/referral to death | Median (IQR): 6 (3-8) | Drugs for acid related disorders (60.8%)  Analgesics (56.7%)  Psycholeptics (53.1%)  Laxatives (51.5%)  Systemic corticosteroids (28.4%) | Not assessed | Assessed |
| McLean 2013 ^[^[^51^](#_ENREF_51)^]^ | Specialist palliative care service comprising an acute hospital and community team | Multiple time points (3 months, 1 month, and 1 week prior to death, and at time of death) | Mean: 10 | Aspirin (42%)  Beta blockers (35 %)  Diuretics (29%)  Statins (29%) | Not assessed | Assessed |
| McNeil 2016 ^[^[^52^](#_ENREF_52)^]^ | Academic and community-based clinical sites that formed part of a clinical trial led by a palliative care research group | Multiple time points (baseline, Week 2, 4, 8, 12, 16, 20, and 24) | Mean (SD): 11.5 (5) | Antihypertensives (70.1%)  Gastric protection (59.8%)  Anti-inflammatories (58.2%)  Laxatives (56.6%) | Not assessed | Assessed |
| Mercadente 2001 ^[^[^53^](#_ENREF_53)^]^ | Home palliative care program | One month before death | 71% (91/128) of patients taking four or more medicines | Ranitidine (51.6%)  Morphine (35.9%)  Methylprednisolone (32.8%)  Diclofenac (29.7%)  Dextropropoxyphene (28.9%) | Not assessed | Not assessed |
| Molist Brunet 2015 ^[^[^54^](#_ENREF_54)^]^ | Acute care unit for older people within a secondary care hospital | Transition to palliative care | Median: 7 | Not specifically reported | Assessed (Study specific patient-centred prescription assessment model for chronic drug therapy) | Assessed |
| Molist Brunet 2014 ^[^[^55^](#_ENREF_55)^]^ | Acute geriatric unit in a secondary care hospital | Admission to discharge | Mean: 7.27 | Not specifically reported | Not assessed | Assessed |
| Nauck 2004 ^[^[^56^](#_ENREF_56)^]^ | Palliative care units | Transition to palliative care | Mean (SD): 3.2 (2.4) | Strong opioids (50.3%)  Non-opioids (42%)  Corticosteroids (17.8%)  Gastric protection (17.6%)  Laxatives (16.3%) | Not assessed | Assessed |
| O’Leary 2018 ^[^[^57^](#_ENREF_57)^]^ | Hospital | No clearly identifiable time point | Mean (SD): 23.32 (0.51) | Not reported | Not assessed | Not assessed |
| Paque 2018 ^[^[^58^](#_ENREF_58)^]^ | Multiple settings that provided palliative care services (hospitals, hospices, nursing home , palliative care home-care service) | Multiple time points (monthly for at least 3 months before death) | Mean (range): 5.67 (0-12) | Opioids (61.8%)  Non-opioid analgesics (59.5%)  Stomach-acid suppressing drugs (57.3%)  Laxatives (56.5%)  Corticosteroids (43.9%) | Not assessed | Assessed |
| Pasina 2018 ^[^[^59^](#_ENREF_59)^]^ | Hospice | Admission/referral to death | Mean (SD): 9.7 (3.4) | Opioids (89.8%)  Systemic corticosteroids (64%)  Hypnotic sedatives (54.3%)  Anxiolytics (46.7%)  Antipsychotics (45.7%) | Assessed (Study specific assessment criteria) | Assessed |
| Pasina 2020 ^[^[^60^](#_ENREF_60)^]^ | Home palliative care program | At transition from to palliative care and before death | Mean (SD): 9.2 (3.6) | Opioids (74.3%)  Systemic corticosteroids (62.6%)  Anxiolytics (40.8%)  Antipsychotics (21.5%) | Assessed (Study specific assessment criteria) | Assessed |
| Raijmakers 2013 ^[^[^61^](#_ENREF_61)^]^ | Hospice* | Last three days of life | Mean (SD): 4.7 (1.8) | Not specifically reported | Assessed (Study specific assessment criteria) | Not assessed |
| Riechelmann 2007 ^[^[^62^](#_ENREF_62)^]^ | Ambulatory palliative care clinic within a hospital | Transition to palliative care | Median (range): 5 (0-21) | Opioids (67%)  Laxatives (54%)  Corticosteroids (41%)  Paracetamol (41%)  Anti-emetics (36%) | Not assessed | Assessed |
| Riechelmann 2009 ^[^[^63^](#_ENREF_63)^,^[^64^](#_ENREF_64)^]^ | Outpatient palliative care clinics clinic within a hospital | Transition to palliative care | Median: 6 (range 0-21) | Opioids (67%)  Laxatives/stool softeners (54%)  Paracetamol (40%)  Corticosteroids (38%)  Anti-dyspeptic agents (36%) | Assessed (Study specific assessment criteria) | Assessed |
| Roux 2019 ^[^[^65^](#_ENREF_65)^]^ | University hospital | Last three months before death | Mean (SD): 6.7 (3.3) | Analgesics (56.4%)  Psycholeptics (51.7%)  Systemic antibacterial agents (23.5%)  Drugs for functional gastrointestinal disorders (21.5%)  Anti-emetics (16.1%) | Assessed (Study specific assessment criteria) | Assessed |
| Russell 2014^[^[^66^](#_ENREF_66)^,^[^67^](#_ENREF_67)^]^ | Hospice and palliative care services | Transition to palliative care | Mean (SD): 7.2 (3.7) | Opioids (29.4%)  Anti-emetics (18.5%)  Benzodiazepines (16.3%)  Laxatives (13.5%)  Paracetamol (9.2%) | Assessed (Beers criteria 2012) | Not assessed |
| Scholes 1995 ^[^[^68^](#_ENREF_68)^]^ | Home care palliative care services | No clearly identifiable time point (cross-sectional assessment) | Median: 4 | Laxatives (50%)  Morphine (28.8%)  NSAIDs (25.4%) | Not assessed | Not assessed |
| Sera 2014a ^[^[^69^](#_ENREF_69)^]^ | Hospices | Transition to palliative care | Mean (range): 16.4 (1-44) | Paracetamol (90.1%)  Lorazepam (86.0%)  Morphine (84.6%)  Atropine (70.3%)  Prochlorperazine (56.0%) | Not assessed | Assessed |
| Sera 2014b ^[^[^70^](#_ENREF_70)^]^ | Hospices | No clearly identifiable time point (cross-sectional assessment) | Mean (range): 15.7 (1-100) | Paracetamol (85.8%)  Lorazepam (84.5%)  Morphine (84.4%)  Atropine (62.5%)  Haloperidol (49.0%) | Not assessed | Not assessed |
| Suhrie 2009 ^[^[^71^](#_ENREF_71)^]^ | Geriatric palliative care unit of a medical care centre | Admission/referral to death | Mean (SD): 9.7 (4.3) | Not reported | Assessed (Study specific assessment tool - Unnecessary Drug Use Measure) | Assessed |
| Tavcar 2014 ^[^[^72^](#_ENREF_72)^]^ | Hospital | Last six days of life | Mean (range): 10.1 (3-15) | Opioids (24%)  Laxatives (17%)  Proton pump inhibitors (13%)  Non-opioids (12%)  Systemic antibiotics/antifungals (5%)  Low molecular weight heparin (5%) | Not assessed | Not assessed |
| Todd 2014 ^[^[^73^](#_ENREF_73)^]^ | Specialist tertiary care palliative care centre | No clearly identifiable time point (cross-sectional assessment) | Mean (range): 12 (1-21) | Not reported | Assessed (Study specific assessment tool (adapted from Holmes et al. 2008)) | Not assessed |
| Toscani 2009 ^[^[^74^](#_ENREF_74)^]^ | Inpatient palliative care units | No clearly identifiable time point (cross-sectional assessment) | Mean (range): 7 (1-15) | Not reported | Not assessed | Not assessed |
| Twycross 1994 ^[^[^75^](#_ENREF_75)^]^ | Palliative care unit within a hospital | Transition to palliative care | Median (range): 5 (0-11) | Morphine (44%)  Co-danthrusate (35%)  Dexamethasone (28%)  Metoclopramide (20%)  Flurbiprofen (19%) | Assessed (Duplicate prescribing) | Assessed |
| Van Nordennen 2016 ^[^[^76^](#_ENREF_76)^]^ | Inpatient palliative care facilities (hospices, palliative care unit in a nursing home) | Admission/referral to death | Mean (SD): 6.1 (3.7) | Analgesics (63.2%)  Drugs for acid related disorders (51.6%)  Psycholeptics (39.4%)  Laxatives (38.7%)  Anticoagulants (33.5%) | Not assessed | Assessed |
| Wenedy 2019 ^[^[^77^](#_ENREF_77)^]^ | Hospice | Day of referral and at death | Mean: 5.9 | Senna glycosides (67%)  Lactulose (59%)  Omeprazole (52.1%)  Morphine (39.6%) | Assessed (OncPal deprescribing guideline) | Assessed |
| West 2014 ^[^[^78^](#_ENREF_78)^]^ | Hospices* | Last three days of life | Mean: 5.95 | Not reported | Assessed (Assessment criteria previously developed by Raijmakers et al. 2013) | Assessed |
| Zueger 2018 ^[^[^79^](#_ENREF_79)^,^[^80^](#_ENREF_80)^]^ | Hospice | Admission/referral to death | Mean (SD): 14.7 (7.2) | Levothyroxine (7.7%)  Furosemide (7.7%)  Morphine (6.7%)  Omeprazole (6.0%)  Potassium chloride (5.5%) | Assessed (Study specific assessment criteria) | Assessed |
| Zueger 2019 ^[^[^81^](#_ENREF_81)^]^ | Hospice | Admission/referral to death | Mean: 14.4 | Not reported | Assessed (Study specific assessment criteria) | Assessed |
